# Bone marrow edema of the femoral head treated with extracorporeal magnetotransduction therapy and conservative management: a case series

**DOI:** 10.1186/s13256-026-06661-0

**Published:** 2026-10-10

**Authors:** Elizabeth Bondi, Martin Ringeisen, Lennart Gerdesmeyer

**Affiliations:** 1 Department of Orthopedics, Mayo Clinic, 200 First St SW, Rochester, MN 55905 USA; 2 Orthopaedic Medical Center Dr. Ringeisen, Konrad-Adenauer Allee 33, 86150 Augsburg, Germany; 3 Department of Orthopaedics and Sports Orthopaedics, Klinikum Rechts Der Isar, Technical University of Munich, Ismaninger Str. 22, 81675 Munich, Germany

**Keywords:** Bone marrow edema, Hip pain, Extracorporeal magnetotransduction therapy, EMTT, Case report, Non-invasive treatment

## Abstract

**Background:**

Bone marrow edema of the hip is a painful condition that can significantly impair function and may lead to more serious joint problems if left untreated. Extracorporeal magnetotransduction therapy (EMTT) is an innovative non-invasive treatment that shows promise in orthopedic applications. However, its use has not been documented for treating bone marrow edema. This case series describes two patients with femoral head bone marrow edema in whom clinical symptoms and radiological findings improved following EMTT given alongside conservative treatment.

**Case presentations:**

We report on two German male patients, aged 44 and 55, who presented with an acute onset of hip pain lasting four weeks, with no history of trauma. Both patients exhibited reduced hip mobility, and magnetic resonance imaging (MRI) scans confirmed bone marrow edema in the femoral head. Each patient underwent eight sessions of EMTT over four weeks (10,000 pulses, 8 Hz, intensity level 8), alongside standard conservative treatments, which included medication, physiotherapy and, in one patient, partial off-loading. At follow-up appointments (13 weeks and 9 weeks post-consultation, respectively), both patients reported complete pain relief and regained their range of motion. MRI results confirmed near-complete resolution of the edema in both cases.

**Conclusion:**

In both patients, symptoms and MRI findings improved following EMTT administered together with conservative treatment, and the treatment was well tolerated. Considering the small number of patients, the concurrent interventions and the self-limiting natural history of bone marrow edema, a causal contribution of EMTT cannot be established from these observations. The favorable course nevertheless supports the formal evaluation of EMTT in this indication, and randomized controlled trials are needed to determine its efficacy in bone marrow edema.

**Supplementary Information:**

The online version contains supplementary material available at https://doi.org/10.1186/s13256-026-06661-0.

## Background

Bone marrow edema (BME) of the hip is a radioclinical diagnosis characterized by increased interstitial fluid in the bone marrow, commonly affecting the femoral head [1]. Patients often experience intense pain and limitations in functioning, which has led to increased interest in BME in recent literature [2]. While BME is a common finding in MRI scans, its exact cause is still not fully understood [3, 4]. It is increasingly recognized not as a standalone diagnosis, but as a radiological sign of an underlying pathology. According to the Ludwig Maximilians University (LMU) Consensus classification, BME can result from various etiologies, including trauma, mechanical overload, inflammation, infection, ischemia, metabolic imbalance, or neoplastic processes [5]. The femoral head is one of the most commonly affected sites, primarily impacting middle-aged men aged 40 to 60 years and pregnant women in their third trimester [6]. BME typically resolves within 6–8 months, with complete healing being the norm [7]. If untreated, BME can lead to progressive conditions such as osteonecrosis of the femoral head, subchondral insufficiency fractures, and/or hip osteoarthritis, which may result in joint destruction, requiring surgical intervention [8]. The conservative treatment involves non or partial weight bearing for 3–6 weeks, pain management with painkillers combined with anti-inflammatory drugs, as well as physical therapy. In addition to pharmacological agents like non-steroidal anti-inflammatory drugs (NSAIDs), the administration of bisphosphonates plays a significant role in the pharmacological management of BME, even though standardized guidelines are lacking [9]. It has also been suggested that extracorporeal shock wave therapy (ESWT) may be a suitable option for conservative treatment in conditions involving BME [10].

Another biophysical therapy modality is electromagnetic wave therapy. Its traditional form, pulsed electromagnetic field therapy (PEMF), has been explored for BME and showed promising results in pain reduction and functional improvement [11]. These electromagnetic fields conventionally operate at frequencies below 100 Hz with magnetic flux densities between 0.1 mT and 30 mT, and most PEMF devices used in bone research generate 1 mT to 10 mT [12, 13]. Extracorporeal magnetotransduction therapy (EMTT) is a novel form, delivering considerably higher physical parameters, with an oscillation frequency of 100 to 300 kHz and a field strength of up to 80 mT [14].

Recent evidence supports the use of EMTT in orthopedic conditions, with randomized controlled trials showing significant pain reduction and functional improvement in patients with chronic back pain, rotator cuff tendinopathy and chronic aseptic osteitis [15–17]. Additionally, case reports suggest EMTT may promote fracture healing [18, 19]. Cellular research indicates that EMTT promotes angiogenesis and enhances osteoblast activity and mineralization, which are crucial for bone repair processes, particularly those related to BME [14, 20, 21].

This case series reports the clinical and radiological course of two patients with BME of the femoral head who received EMTT in addition to conservative treatment.

## Case presentations

### Patient 1

**Fig. 1:**
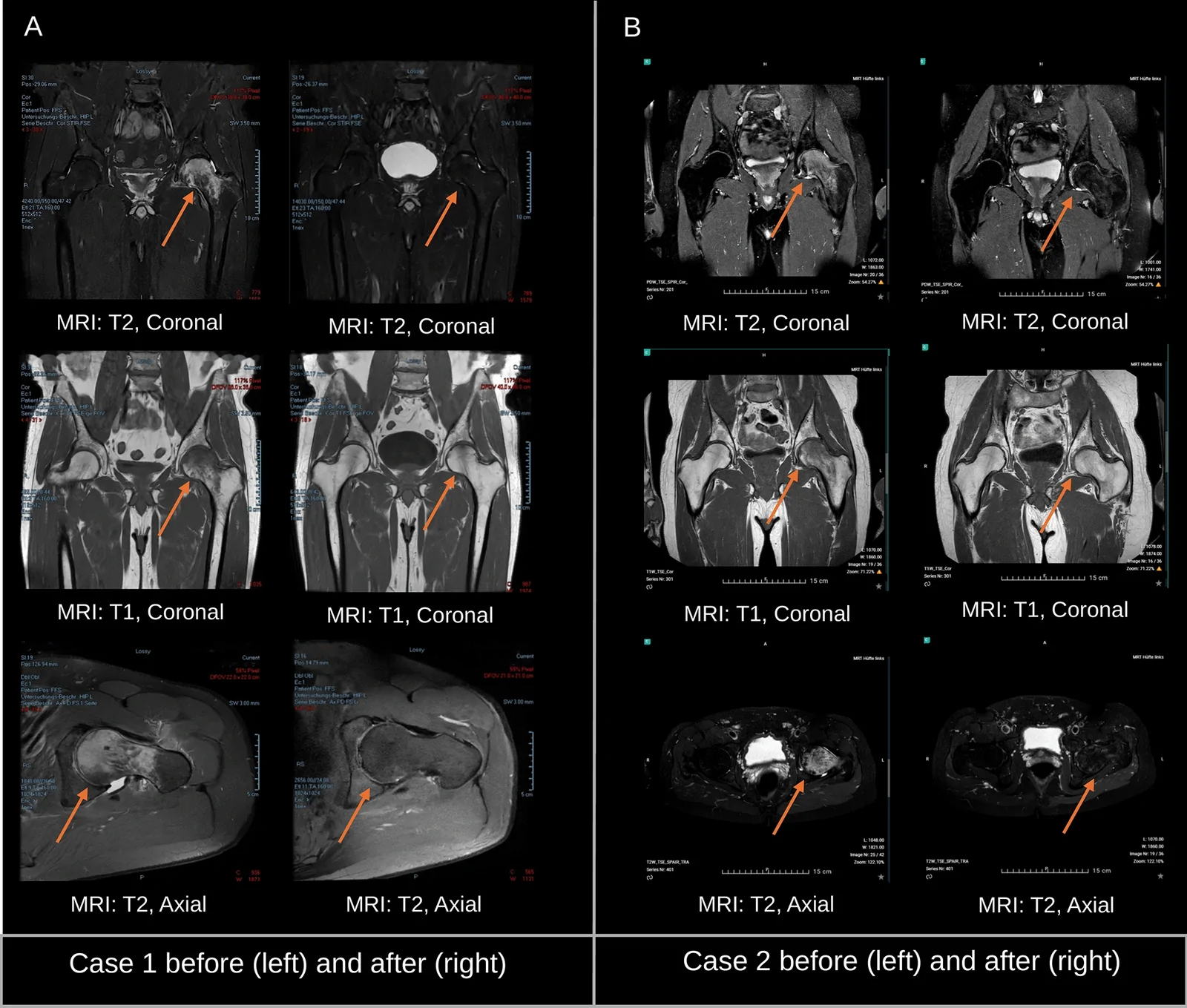
Magnetic resonance imaging (MRI) of the left hip before and after extracorporeal magnetotransduction therapy (EMTT). **A** Case 1, **B** Case 2. For each case the left column shows the study at initial presentation and the right column the follow-up study. Rows show T2-weighted coronal (top), T1-weighted coronal (middle) and T2-weighted axial (bottom) images. Arrows indicate the left femoral head. In Case 1 the initial study shows extensive bone marrow edema of the left femoral head, hyperintense on T2-weighted and hypointense on T1-weighted images. The edema is diffuse and confluent, without a demarcated band-shaped lesion. At follow-up, 13 weeks after initial presentation, the edema is markedly reduced and marrow signal has largely normalized. In Case 2 the initial study shows bone marrow edema of the left femoral head with corresponding signal characteristics. At follow-up, 9 weeks after initial presentation, the edema has nearly resolved

A 55-year-old German male, an amateur footballer and recreational runner, presented with a 4-week history of left hip pain without any preceding trauma. His medical history included diabetes mellitus and the placement of a coronary stent. Physical examination revealed pain on flexion and internal rotation of the left hip, with a range of motion of flexion/extension 50°/0/10°, internal/external rotation 5°/0/30° and abduction/adduction 30°/0/5°. MRI of the left hip showed extensive BME of the femoral head. The edema was diffuse and confluent rather than band-shaped and geographically demarcated, and no subchondral low-signal line running parallel to the articular surface was present. These features argue against an early osteonecrotic lesion and against a subchondral insufficiency fracture, and support the diagnosis of bone marrow edema syndrome [1, 5]. The initial radiological report raised the suspicion of femoral head necrosis and described an early subchondral impression, neither of which was confirmed on review or on follow-up imaging. The working diagnosis was therefore bone marrow edema syndrome of the femoral head. The patient underwent eight sessions of EMTT over four weeks using the Magnetolith device with its standard applicator (STORZ Medical AG, Tägerwilen, Switzerland). The stimulation protocol was set to level 8, corresponding to a magnetic field strength of approximately 80 mT, with a pulse repetition rate of 8 Hz and 10,000 pulses per session (~ 21 minutes). The pulse repetition rate of 8 Hz refers to the number of magnetic pulses delivered per second and is distinct from the oscillation frequency of 100 to 300 kHz within each individual pulse. With the patient lying supine, the EMTT applicator was positioned over the anterolateral aspect of the affected hip, centered over the projection of the femoral head. Applicator position was kept constant across all eight sessions. Treatment was delivered through clothing without direct skin contact and without coupling medium. The patient experienced mild discomfort that lasted 1–2 hours after each session. Each session was followed by progressive functional improvement. Concomitant treatment consisted of celecoxib 200 mg orally as required for analgesia from initial presentation, later changed to ibuprofen 600 mg as required, and physiotherapy for mobilization, six sessions once weekly, prescribed at the end of the EMTT course in week five. No off-loading was prescribed. Post-treatment, he reported significant pain relief and improved hip function. A follow-up MRI 6 weeks after the initial consultation showed partial regression of the edema and, in addition, early osteoporotic changes of the hip in side-to-side comparison. Oral ibandronate 150 mg monthly was therefore started for fracture prophylaxis rather than for treatment of the edema. It was continued as long-term osteoporosis prophylaxis and was ongoing at the time of last follow-up. Enoxaparin 4000 IU subcutaneously was given for a short period at week 15 as post-operative thromboprophylaxis following unrelated hand surgery. A second follow-up MRI at 13 weeks after the initial consultation demonstrated regression of the edema (Fig. 1, A). At that point, his range of motion had improved to flexion/extension 100°/0/10°, internal/external rotation 20°/0/40° and abduction/adduction 45°/0/20°, indicating substantial restoration of joint function.

### Patient 2

A 44-year-old German male, an IT professional and amateur footballer, presented with acute left hip pain of four weeks' duration and gradually worsening pain. He had no history of trauma or alcohol use. He had experienced the same condition in the right hip in 2014, which had resolved. Physical examination revealed reduced mobility of the left hip with flexion/extension 50°/0/5°, internal/external rotation 5°/0/30° and abduction/adduction 20°/0/0. MRI showed BME of the left femoral head without signs of necrosis. The patient received eight sessions of EMTT over four weeks. The stimulation protocol, similar to that of patient 1, was set at level 8, corresponding to approximately 80 mT, with a pulse repetition rate of 8 Hz and a total of 10,000 pulses per session (~ 21 minutes). Applicator positioning was likewise standardized, with the applicator placed anterolaterally over the projection of the femoral head and held in the same position throughout the treatment course. No signs of discomfort were observed. Concomitant treatment consisted of partial off-loading for six weeks and physiotherapy for mobilization, six sessions once weekly, both from initial presentation, enoxaparin 4000 IU subcutaneously daily for thromboprophylaxis during the six weeks of off-loading, and oral ibandronate 150 mg monthly, started two weeks after initial presentation and continued throughout the EMTT course. In this patient early osteoporotic changes were already apparent on the initial MRI, and the bisphosphonate was started for fracture prophylaxis rather than for treatment of the edema. It was continued as long-term osteoporosis prophylaxis and was ongoing at the time of last follow-up. Post-treatment, he experienced marked pain reduction and improved hip mobility with the following post-treatment hip range of motion: flexion/extension 110°/0/10°, internal/external rotation 15°/0/45° and abduction/adduction 40°/0/15°. A follow-up MRI at 9 weeks after the first consultation demonstrated near-complete resolution of the edema (Fig. 1B).

## Discussion and conclusions

The two patients presented demonstrate a typical clinical course of BME, with intensifying pain between the second and third months after initial presentation, making weight-bearing challenging and limiting the patients' range of motion. Normally, BME resolves within six to eight months [7]. Alongside standard treatment with medication and physiotherapy, our patients received EMTT twice a week for four consecutive weeks. The follow-up for the first patient took place at 13 weeks after the initial consultation and 9 weeks for the second patient, when the follow-up MRIs were performed. At follow-up both patients were pain free, weight bearing fully and had regained range of motion, and MRI showed near-complete resolution of the edema.

Research indicates that EMTT positively affects bone health disorders. Several case reports have demonstrated improved fracture healing and successful treatment of non-unions [18, 19]. Experimental studies suggest that EMTT enhances angiogenesis and osteogenesis in mesenchymal stem cells by increased vascular endothelial growth factors (VEGF), which are essential factors in bone regeneration [14, 22]. Recent cellular research has shown that EMTT promotes bone formation by promoting the differentiation and maturation of osteoblasts, as well as improving the mineralization of the bone matrix [20]. The pathophysiology of BME remains not fully understood and is a topic of uncertainty. BME lesions exhibit increased vascularity and angiogenesis, suggesting a need for repair processes [23]. Histomorphological analyses have indicated decreased bone mineralization, which may elucidate the subtle and transient radiolucency observed in affected femoral heads [21]. These researchers propose that active bone formation is essential for the self-limiting and reversible nature of most BME cases. Considering the documented effects of EMTT in stimulating both osteogenesis and angiogenesis, its application has been proposed as a means of supporting the natural healing process in BME. The present observations are consistent with this hypothesis, although prospective studies are required to test it. Whether early intervention with EMTT can influence the progression of BME to conditions that require surgery, such as osteonecrosis, subchondral insufficiency fracture or hip osteoarthritis, is an open question that merits prospective investigation [8].

Several limitations apply to this report. It describes an uncontrolled series of two patients who received EMTT alongside conservative treatment, which in both patients included a bisphosphonate started during or shortly after the EMTT course and continued thereafter. The contribution of EMTT can therefore not be separated from that of the concomitant interventions or from the natural course of BME. No validated outcome measures were applied, so the reported improvement rests on patient-reported pain and on clinically assessed range of motion. Follow-up intervals differed between the two patients and followed routine clinical scheduling rather than a predefined protocol.

These limitations can be addressed in prospective controlled studies using validated outcome measures and predefined follow-up intervals. A systematic review demonstrated that ESWT significantly enhances pain relief and functional ability, indicating it as an effective treatment for BME [10]. Combining ESWT and EMTT may result in synergistic effects for treating musculoskeletal disorders, such as non-union and rotator cuff tendinopathy [16, 18]. Future research should explore the combination of ESWT and EMTT as a treatment option for BME.

## Conclusion

This case series reports promising clinical and radiological findings following EMTT in patients with BME of the femoral head, with pain relief, functional recovery and regression of the edema observed in both patients. As an uncontrolled series of two patients treated alongside conservative measures, these findings require confirmation in randomized controlled studies, which should also define the role of EMTT in the treatment of BME and its potential combination with ESWT.

## Supplementary Information

Supplementary Material 1.

## Data Availability

All data supporting the findings of this study are available within the paper.

## Abbreviations

BME: Bone marrow edema
EMTT: Extracorporeal magnetotransduction therapy
ESWT: Extracorporeal shock wave therapy
MRI: Magnetic resonance imaging
NSAIDs: Non-steroidal anti-inflammatory drugs
PEMF: Pulsed electromagnetic field therapy
T1/T2: T1-weighted / T2-weighted MRI sequences
