# Supplementary material for: Bone marrow edema of the femoral head treated with extracorporeal magnetotransduction therapy and conservative management: a case series
Source: J Med Case Rep. 2026 Oct 10;20:613. doi: 10.1186/s13256-026-06661-0 (PMC13654217; doi:10.1186/s13256-026-06661-0)
Supplement: Supplementary file 1 [file 13256_2026_6661_MOESM1_ESM.pdf]

CARE Checklist of information to include when writing a case report

CARE case report guidelines

(cc) BY-NC-ND

Topic	Item	Checklist item description	Reported on Line
Title	1	The diagnosis or intervention of primary focus followed by the words "case report"	Title
Key Words	2	2 to 5 key words that identify diagnoses or interventions in this case report, including "case report"	27,28
Abstract (no references)	3a	Introduction: What is unique about this case and what does it add to the scientific literature?	4-8
	3b	Main symptoms and/or important clinical findings	11-13
	3c	The main diagnoses, therapeutic interventions, and outcomes	13-18
	3d	Conclusion—What is the main "take-away" lesson(s) from this case?	21-24
Introduction	4	One or two paragraphs summarizing why this case is unique (**may include references**)	57-58
Patient Information	5a	De-identified patient specific information.	61-63; 81-82
	5b	Primary concerns and symptoms of the patient.	61-64; 81-84
	5c	Medical, family, and psycho-social history including relevant genetic information	61-63; 81-82
	5d	Relevant past interventions with outcomes	N/A
Clinical Findings	6	Describe significant physical examination (PE) and important clinical findings.	63-65; 83-85
Timeline	7	Historical and current information from this episode of care organized as a timeline	61-62; 81-82
Diagnostic Assessment	8a	Diagnostic testing (such as PE, laboratory testing, imaging, surveys).	64; 84-85
	8b	Diagnostic challenges (such as access to testing, financial, or cultural)	N/A
	8c	Diagnosis (including other diagnoses considered)	65-66; 84-85
	8d	Prognosis (such as staging in oncology) where applicable	N/A
Therapeutic Intervention	9a	Types of therapeutic intervention (such as pharmacologic, surgical, preventive, self-care)	66-69; 85-88
	9b	Administration of therapeutic intervention (such as dosage, strength, duration)	66-69; 85-88
	9c	Changes in therapeutic intervention (with rationale)	N/A
Follow-up and Outcomes	10a	Clinician and patient-assessed outcomes (if available)	69-73; 88-91
	10b	Important follow-up diagnostic and other test results	70-71; 90-91
	10c	Intervention adherence and tolerability (How was this assessed?)	68; 86-87
	10d	Adverse and unanticipated events	68
Discussion	11a	A scientific discussion of the strengths AND limitations associated with this case report	101-125
	11b	Discussion of the relevant medical literature **with references**.	108-117; 120-125
	11c	The scientific rationale for any conclusions (including assessment of possible causes)	104-107
	11d	The primary "take-away" lessons of this case report (without references) in a one paragraph conclusion	127-131
Patient Perspective	12	The patient should share their perspective in one to two paragraphs on the treatment(s) they received	68; 86-87
Informed Consent	13	Did the patient give informed consent? Please provide if requested	Yes ☒ No ☐
